# Identification of a Regulatory Variant That Binds FOXA1 and FOXA2 at the *CDC123/CAMK1D* Type 2 Diabetes GWAS Locus

**DOI:** 10.1371/journal.pgen.1004633

**Published:** 2014-09-11

**Authors:** Marie P. Fogarty, Maren E. Cannon, Swarooparani Vadlamudi, Kyle J. Gaulton, Karen L. Mohlke

**Affiliations:** 1 Department of Genetics, University of North Carolina, Chapel Hill, Chapel Hill, North Carolina, United States of America; 2 Wellcome Trust Centre for Human Genetics, University of Oxford, Oxford, United Kingdom; Harvard Medical School, United States of America

## Abstract

Many of the type 2 diabetes loci identified through genome-wide association studies localize to non-protein-coding intronic and intergenic regions and likely contain variants that regulate gene transcription. The *CDC123/CAMK1D* type 2 diabetes association signal on chromosome 10 spans an intergenic region between *CDC123* and *CAMK1D* and also overlaps the *CDC123* 3′UTR. To gain insight into the molecular mechanisms underlying the association signal, we used open chromatin, histone modifications and transcription factor ChIP-seq data sets from type 2 diabetes-relevant cell types to identify SNPs overlapping predicted regulatory regions. Two regions containing type 2 diabetes-associated variants were tested for enhancer activity using luciferase reporter assays. One SNP, rs11257655, displayed allelic differences in transcriptional enhancer activity in 832/13 and MIN6 insulinoma cells as well as in human HepG2 hepatocellular carcinoma cells. The rs11257655 risk allele T showed greater transcriptional activity than the non-risk allele C in all cell types tested. Using electromobility shift and supershift assays we demonstrated that the rs11257655 risk allele showed allele-specific binding to FOXA1 and FOXA2. We validated FOXA1 and FOXA2 enrichment at the rs11257655 risk allele using allele-specific ChIP in human islets. These results suggest that rs11257655 affects transcriptional activity through altered binding of a protein complex that includes FOXA1 and FOXA2, providing a potential molecular mechanism at this GWAS locus.

## Author Summary

GWAS have identified more than 1200 loci contributing to risk of disease, including more than 70 loci associated with type 2 diabetes. With a majority of associated variants localized to non-coding regions of the genome, focus has moved to identifying the functional variants explaining the association signals. One mechanism by which variants may act is to affect activity of enhancer elements regulating target gene expression. In this study, we take advantage of recent advances in genome-wide annotation of human regulatory elements to prioritize candidate functional variants at the *CDC123*/*CAMK1D* locus. We identify two T2D-associated variants that overlap predicted regulatory enhancer elements. We demonstrate that one variant, rs11257655, shows allele-specific transcriptional enhancer activity in mammalian cell lines relevant to type 2 diabetes. We also show differential protein-DNA binding suggesting that the rs11257655 type 2 diabetes- risk allele increased transcriptional activity through binding a protein complex that includes FOXA1 and FOXA2. This study demonstrates that genome-wide maps of regulatory elements are a useful resource to guide identification of variants differentially affecting transcriptional activity and provides insight into molecular mechanisms underlying a T2D susceptibility locus.

## Introduction

Type 2 diabetes is a complex metabolic disease with a substantial heritable component [1]. Over the past seven years, genome-wide association studies (GWAS) have successfully identified over 70 common risk variants associated with type 2 diabetes [2]–[5]. Association signals at many of these loci localize to non-protein-coding intronic and intergenic regions and likely harbor regulatory variants altering gene transcription. In recent years great advances have facilitated identification of regulatory elements genome-wide using techniques including DNase-seq and FAIRE-seq (formaldehyde-assisted isolation of regulatory elements), which identify regions of nucleosome depleted open chromatin, and ChIP-seq (chromatin immunoprecipitation), which identify histone modifications to nucleosomes and transcription factor binding sites. Several studies have successfully integrated trait-associated variants at GWAS loci with publicly available regulatory element datasets in disease-relevant cell types to guide identification of regulatory variants underlying disease susceptibility [6]–[10].

The *CDC123* (cell division cycle protein 123)/*CAMK1D* (calcium/calmodulin-dependent protein kinase ID) locus on chromosome 10 contains common variants (MAF>.05) strongly associated with type 2 diabetes in Europeans (rs12779790, *P* = 1.2×10^−10^) [3], East Asians (rs10906115, *P* = 1.5×10^−8^) [4], and South Asians (rs11257622, *P* = 5.8×10^−6^) [5]. Fine-mapping using the Metabochip identified rs11257655 as the lead SNP [2]. The index variant and proxies (r^2^>.7) span an intergenic region of at least 45 kb between *CDC123* and *CAMK1D* and overlap the 3′ end of *CDC123* [3]. None of the type 2 diabetes-associated variants at this locus are located in exons. Analysis of the beta cell function measurements HOMA-B and insulinogenic index, derived from paired glucose and insulin measures at fasting or 30 minutes after a glucose challenge, demonstrated association of the risk allele at the *CDC123/CAMK1D* locus with reduced beta cell function, suggesting the beta cell as a candidate affected tissue [2], [11]. Another intronic variant (rs7068966, r^2^ = 0.18 EUR, 1000G Phase 1) located 50 kb away from rs12779790 is associated with lung function [12].

The transcript(s) targeted by risk variant activity at this locus remain unknown. *CDC123* is regulated by nutrient availability in yeast and is essential to the onset of mRNA translation and protein synthesis through assembly of the eukaryotic initiation factor 2 complex [13], [14]. Evidence from previous GWA studies suggest cell cycle dysregulation as a common mechanism in type 2 diabetes; for example, type 2 diabetes association signals are found close to the cell cycle regulator genes, CDKN2A/CDKN2B and *CDKAL1* [15]. *CAMK1D* is a member of the Ca^2+^/calmodulin-dependent protein kinase family which transduces intracellular calcium signals to affect diverse cellular processes. Upon calcium influx in granulocyte cells and hippocampal neurons, CAMK1D activates CREB-dependent gene transcription [16], [17]. Given the roles of cytosolic calcium in regulation of beta cell exocytotic machinery and of *CREB* in beta cell survival, *CAMK1D* may have a role in beta cell insulin secretion. In *cis*-eQTL analyses, the rs11257655 type 2 diabetes risk allele was more strongly and directly associated with increased expression of *CAMK1D* than *CDC123* in both blood and lung [18], [19].

In this study we aimed to identify the variant(s) underlying the association signal at the *CDC123*/*CAMK1D* locus using genome-wide maps of open chromatin, chromatin state and transcription factor binding in pancreatic islets, hepatocytes, adipocytes and skeletal muscle myotubes. We measured transcriptional activity of variants in putative regulatory elements using luciferase reporter assays, and identified a candidate *cis*-acting SNP driving allele-specific enhancer activity in two mammalian beta cell-lines as well as hepatocellular carcinoma cells. We then evaluated DNA-protein binding in sequence surrounding this variant and identified allele-specific binding to key islet and hepatic transcription factors. Thus, our study provides strong evidence of a functional variant underlying the type 2 diabetes association signal at the *CDC123/CAMK1D* locus acting through altered regulation in type 2 diabetes-relevant cell types.

## Results

### Prioritization of type 2 diabetes-associated SNPs with regulatory potential at the *CDC123/CAMK1D* locus

**Figure 1:**
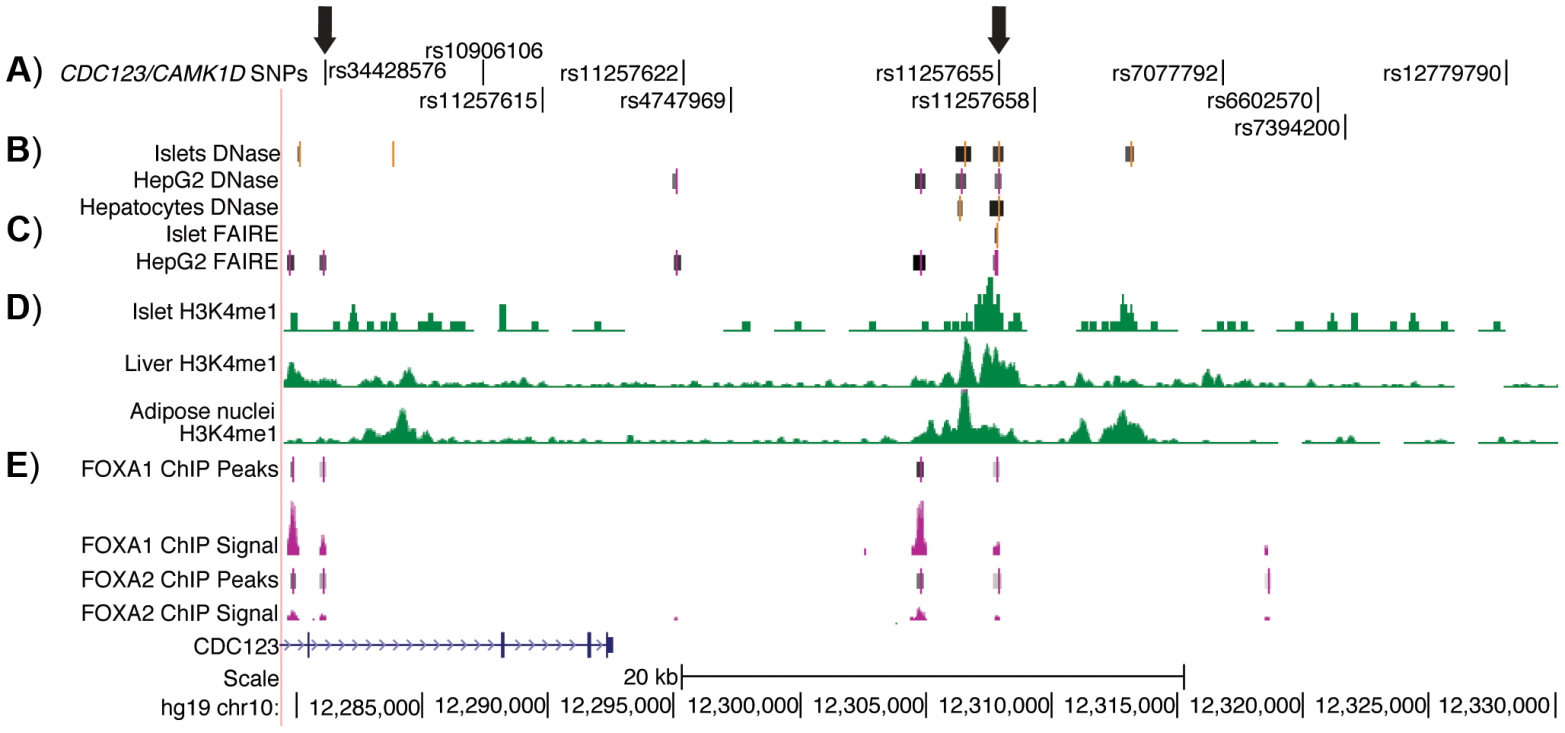
Regulatory potential at type 2 diabetes-associated SNPs at the *CDC123/CAMK1D* locus. A) The 11 SNPs in high LD (r^2^≥.7, EUR) with GWAS index SNP rs12779790. Arrows indicate the two SNPs that overlap islet, liver, and HepG2 open chromatin and epigenomic marks and that are located near to HepG2 ChIP-seq peaks; these two SNPs were tested for allele-specific transcriptional activity. B) DNase hypersensitivity peaks identified in two pooled islet samples from the ENCODE Consortium. C) FAIRE peaks identified in one representative islet sample from the ENCODE Consortium. D) H3K4me1 histone modifications from the Roadmap Epigenomics Consortium. E) FOXA1 and FOXA2 ChIP-seq peaks and signal from ENCODE. Image is taken from the UCSC genome browser, February 2009 (GRCh37/hg19) assembly (http://genome.ucsc.edu) [51]. The 5′ end of *CAMK1D* begins after position 12,390,000.

To identify potentially functional SNPs at the *CDC123/CAMK1D* locus, we considered variants in high LD (r^2^≥.7, EUR, 1000G Phase 1 release) with GWAS index SNP rs12779790. To further prioritize variants for functional follow up, we used genome wide maps of chromatin state (Figure 1) in available type 2 diabetes-relevant cell types including pancreatic islets, liver hepatocytes, skeletal muscle myotubes and adipose nuclei. Variant position was evaluated with respect to DNase- and FAIRE-seq peaks and several histone modifications, including H3K4me1 and H3K9ac. DNase and FAIRE are established methods of identification of nucleosome depleted regulatory regions [20], while H3K4me1 and H3K9ac are post-translational chromatin marks often associated with enhancer regions [21], [22]. We also assessed chromatin occupancy by transcription factors using available genome wide ChIP-seq data sets. Of 11 variants meeting the LD threshold, two SNPs were found to overlap chromatin signals. One SNP, rs11257655 (r^2^ = .74 with GWAS index SNP rs12779790), located 15 kb from the 3′ end of *CDC123* and 84 kb from the 5′ end of *CAMK1D*, was a particularly plausible candidate overlapping islet, liver and HepG2 cell line DNase peaks, islet and liver FAIRE peaks, H3K4me1 and H3K9ac chromatin marks, and FOXA1 and FOXA2 ChIP-seq peaks in HepG2 cells (Figure S1). A second SNP, rs34428576 (r^2^ = .71 with rs12779790), overlapped a HepG2 DNase peak and displayed occupancy by FOXA1 and FOXA2 binding in HepG2 cells (Figure 1). No SNPs overlapped with DNase peaks in skeletal muscle myotubes.

### Allele-specific enhancer activity of rs11257655 in islet and liver cells

**Figure 2:**
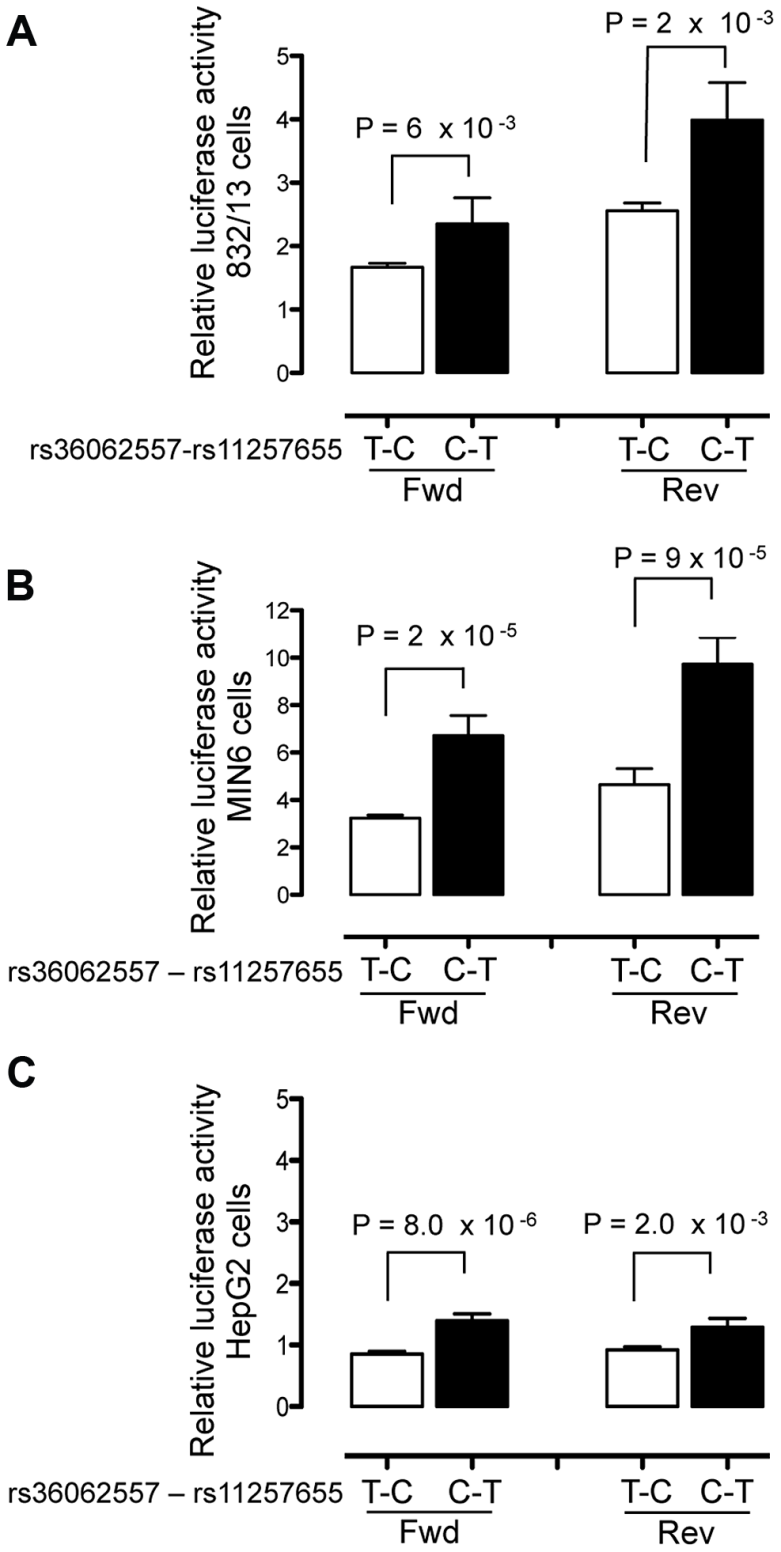
Haplotype containing type 2 diabetes-associated SNPs displays differential transcriptional activity. Enhancer activity was tested in 832/13, MIN6 and HepG2 cells for the type 2 diabetes non-risk (white bars) and risk (black bars) haplotypes in the forward and reverse orientations with respect to the genome. Risk refers to the rs11257655 variant; rs36062557 is included in the haplotype due to proximity. The haplotype containing risk allele rs11257655-T shows greater transcriptional activity than the non-risk allele rs11257655-C in both orientations with respect to a minimal promoter vector in 832/13 cells (A), MIN6 cells (B) and HepG2 cells (C). Error bars represent standard deviation of 4–5 independent clones for each allele. Firefly luciferase activity was normalized to *Renilla* luciferase activity, and normalized results are expressed as fold change compared to empty vector control. *P* values were calculated by a two-sided *t*-test.

To evaluate transcriptional activity of the SNPs in predicted regulatory regions, 150–200 bp surrounding each SNP allele was cloned into a minimal promoter vector and luciferase activity was measured in two beta cell lines, 832/13 rat insulinoma and MIN6 mouse insulinoma cells, and in HepG2 liver hepatocellular carcinoma cells. Four to five independent clones for each allele were generated and enhancer activity was measured in duplicate for each clone. A 151-bp region including rs11257655 (and rs36062557 due to proximity, r^2^ = .38 with rs11257655) showed differential allelic enhancer activity in both orientations in all three cell lines (Figure 2). The risk allele rs11257655-T showed significantly increased luciferase activity compared to the non-risk allele rs11257655-C (forward: 832/13 *P* = 6.3×10^−3^, MIN6 *P* = 1.7×10^−5^; HepG2 *P* = 8.0×10^−5^; reverse: 832/13 *P* = 2.2×10^−3^, MIN6 *P* = 9.9×10^−5^; HepG2 *P* = 2.0×10^−3^). Enhancer activity represents greater than a 1.4-fold (HepG2, MIN6) to 2.1-fold (832/13) increase in transcriptional activity relative to the non-risk allele in both the forward and reverse orientations. Compared to an empty vector control, enhancer activity was greatest in the islet cell lines (risk allele: 832.13, 4-fold; MIN6, 10-fold; HepG2, 1.6-fold).

A 179-bp region surrounding the second candidate SNP rs34428576 showed only moderate allele-specific activity, and only in the reverse orientation, in HepG2 cells (*P = *.02) and no allele-specific activity in islet cells (Figure S2).

**Figure 3:**
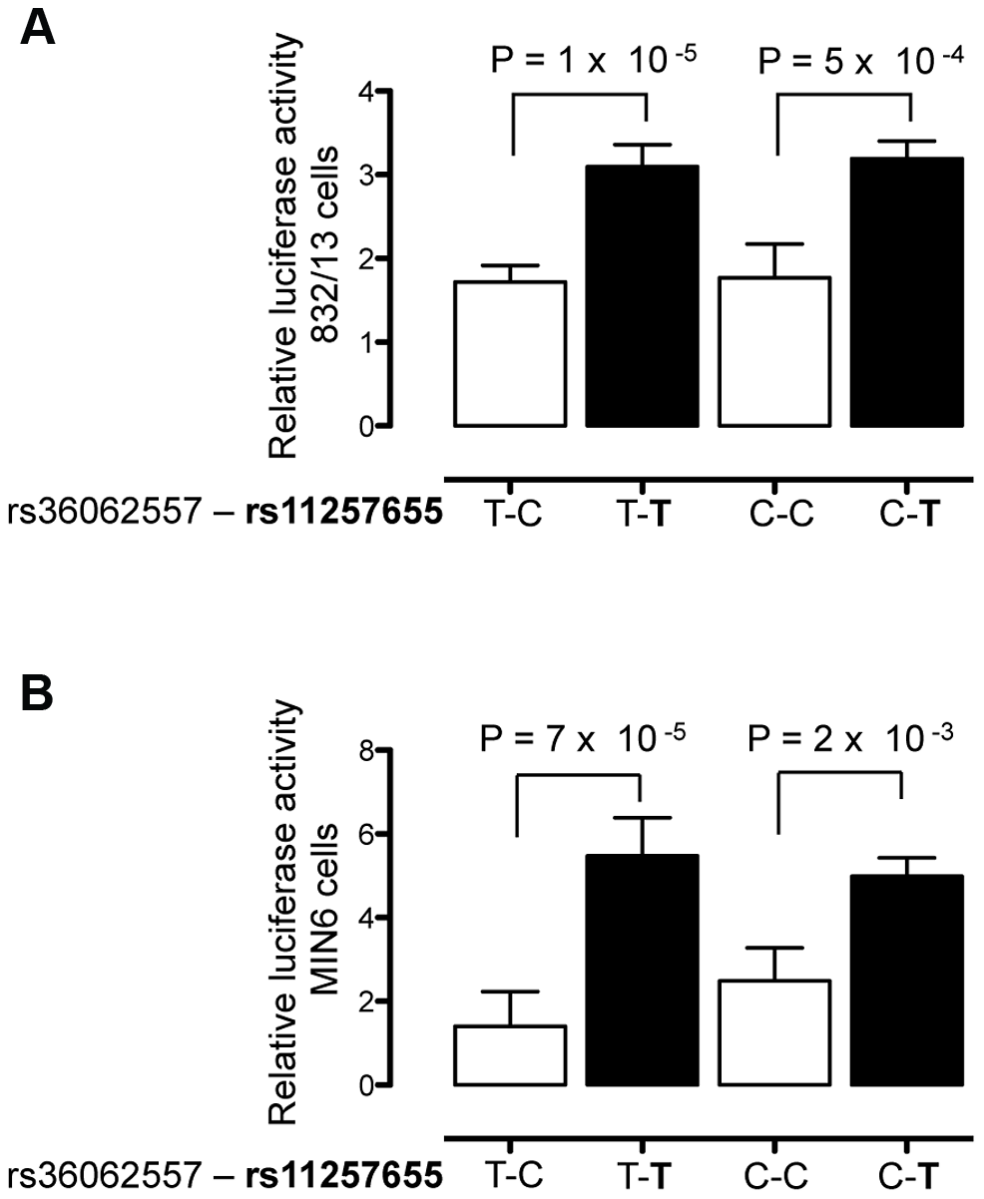
rs11257655 drives differential transcriptional activity. Site-directed mutagenesis was carried out to separate the effects of rs36062557 from rs11257655. Enhancer activity was tested in 832/13 and MIN6 and cells for the type 2 diabetes non-risk (white bars) and risk (black bars) haplotypes in the forward orientation. The risk allele rs11257655-T shows greater transcriptional activity than the non-risk allele rs11257655-C independent of rs36062557 genotype in 832/13 cells (A) and MIN6 cells (B). Error bars represent standard deviation of 2–4 independent clones for each allele. Results are expressed as fold change compared to empty vector control. *P* values were calculated by a two-sided *t*-test.

To verify that rs11257655 and not rs36062557 accounted for allele-specific effects, we used site-directed mutagenesis to construct the remaining haplotype combinations. The T risk allele of rs11257655 exhibited >1.8 fold increased transcriptional activity compared to the non-risk allele C independent of rs36062557 genotype (Figure 3A, B). In contrast, altering alleles of rs36062557 on a consistent rs11257655 background showed no significant effect on transcriptional activity. Taken together, these data confirm that rs11257655 exhibits allelic differences in transcriptional enhancer activity and suggest it functions within a *cis-*regulatory element at the *CDC123/CAMK1D* type 2 diabetes-associated locus.

### Alleles of rs11257655 differentially bind FOX transcription factors

**Figure 4:**
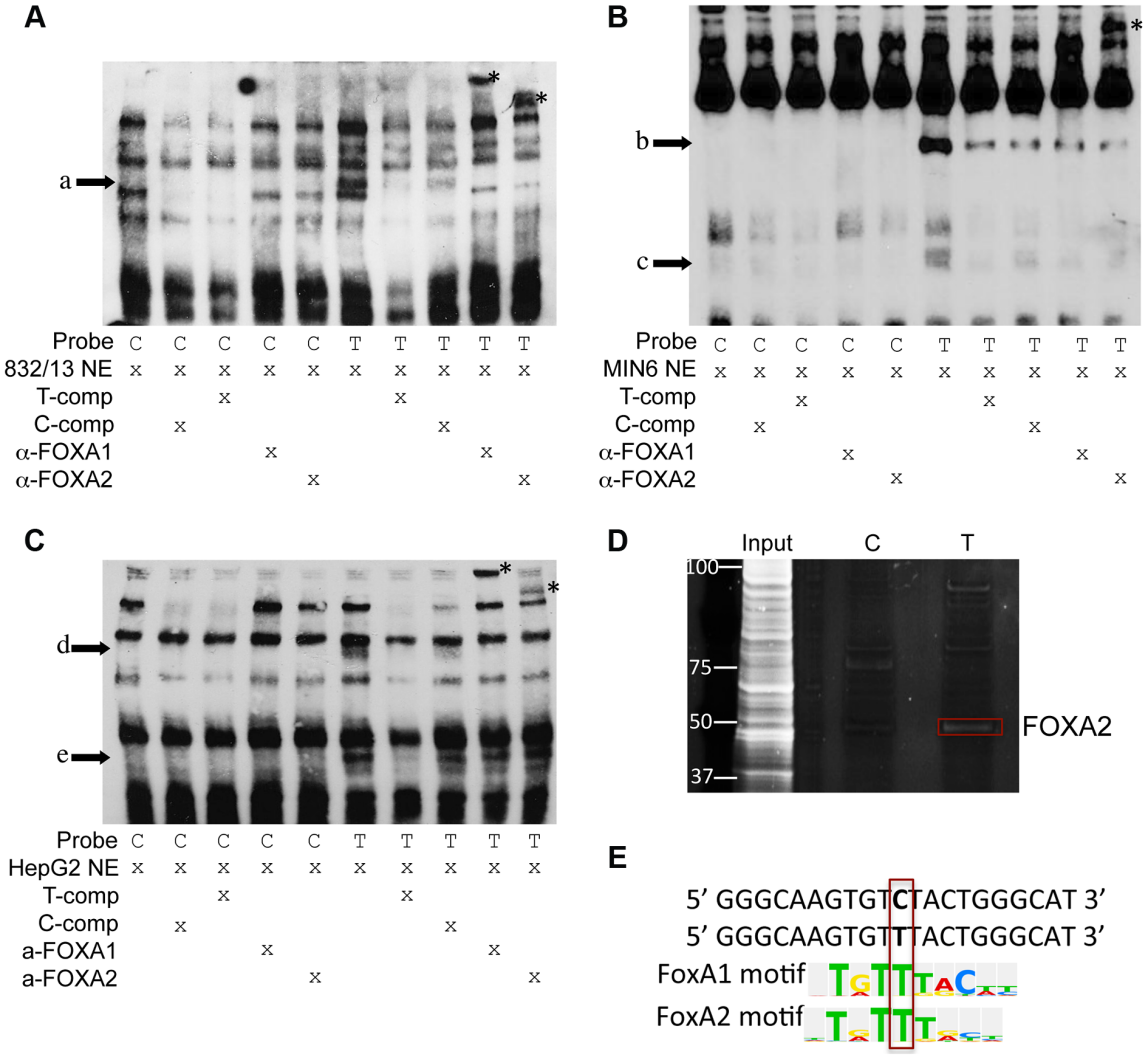
Alleles of rs11257655 differentially bind FOXA proteins in rat 832/13 insulinoma cells, mouse MIN6 insulinoma cells and human HepG2 hepatoma cells. EMSA using 832/13 (A), MIN6 (B) and HepG2 (C) nuclear extract shows differential protein-DNA binding of rs11257655 alleles. The probe containing risk allele rs11257655 -T shows allele-specific protein binding (arrows a–e) compared to the probe containing non-risk allele C. Excess unlabeled probe containing the T allele (T-comp) more efficiently competed away allele-specific bands than unlabeled probe for the C allele (C-comp). Incubation of 832/13 and HepG2, nuclear extract with FOXA1/FOXA2 antibodies disrupt the DNA-protein complex formed with T allele-containing DNA probe (arrow a, d, e) and result in band supershifts (asterisks). Incubation of MIN6 nuclear extract with FOXA2 antibody decreases the DNA-protein complex formed with T allele-containing DNA probe (arrow b) and results in a band supershift. To enhance visualization of protein complexes, free biotin-labeled probe is not shown. (D) DNA affinity-capture identified differential binding of FOXA2 at rs11257655 alleles in 832/13 cells. (E) The T allele of rs11257655 is predicted as a FOXA1 and FOXA2 consensus core-binding motif.

To assess whether alleles of rs11257655 differentially affect protein-DNA binding *in vitro*, biotin-labeled probes surrounding the T (risk) or C (non-risk) allele were incubated with 832/13, MIN6 or HepG2 nuclear lysate and subjected to electrophoretic mobility shift assays (EMSA). Band shifts indicative of multiple DNA-protein complexes were observed for both rs11257655 alleles (Figure 4A, 4B, 4C). In EMSAs from all three cell nuclear extracts, protein complexes were observed for the probe containing the T allele that were not present for the probe containing the C allele (832/13, arrow a; MIN6, arrows b, c, d; HepG2, arrows e, f) suggesting differential protein binding dependent on the rs11257655 allele. Competition of labeled T-allele probe with excess unlabeled T-allele probe more efficiently competed away allele-specific bands than excess unlabeled C-allele probe, demonstrating allele-specificity of the protein-DNA complexes (Figure 4A, 4B, 4C). rs11257655 did not show a differential protein binding pattern in EMSA using 3T3-L1 mouse adipocytes. To examine transcription factor binding to rs11257655, we used a DNA-affinity capture assay. We observed one protein band showing allele-specific binding to the T allele (Figure 4D) that was identified as transcription factor FOXA2 using MALDI TOF/TOF mass spectrometry.

A search in the JASPAR CORE database provided further evidence that the rs11257655 SNP is located within predicted binding sites for FOXA1 and FOXA2, with only the T risk-allele predicted to contain a FOXA1 and FOXA2 consensus core-binding motif (Figure 4E) [23]. To assess binding to FOXA1 and FOXA2, we performed supershift experiments incubating DNA-protein complexes with antibodies for these factors. Incubation of the T allele-protein complex with FOXA1 antibody resulted in a band supershift in 832/13 and HepG2 cells (asterisk, Figure 4A, 4C) A FOXA2-mediated supershift was observed in 832/13, MIN6 and HepG2 cells (asterisk, Figure 4A, 4B, 4C). Differences in antibody species reactivity may account for the lack of a visible FOXA1-mediated supershift in MIN6 cells. Collectively, these results suggest that rs11257655 is located in binding sites for a transcriptional regulator complex including FOXA1 and/or FOXA2, which bind preferably to the rs11257655-T allele in beta cell and liver cell lines.

### FOXA1 and FOXA2 occupancy at rs11257655 in human islets

**Figure 5:**
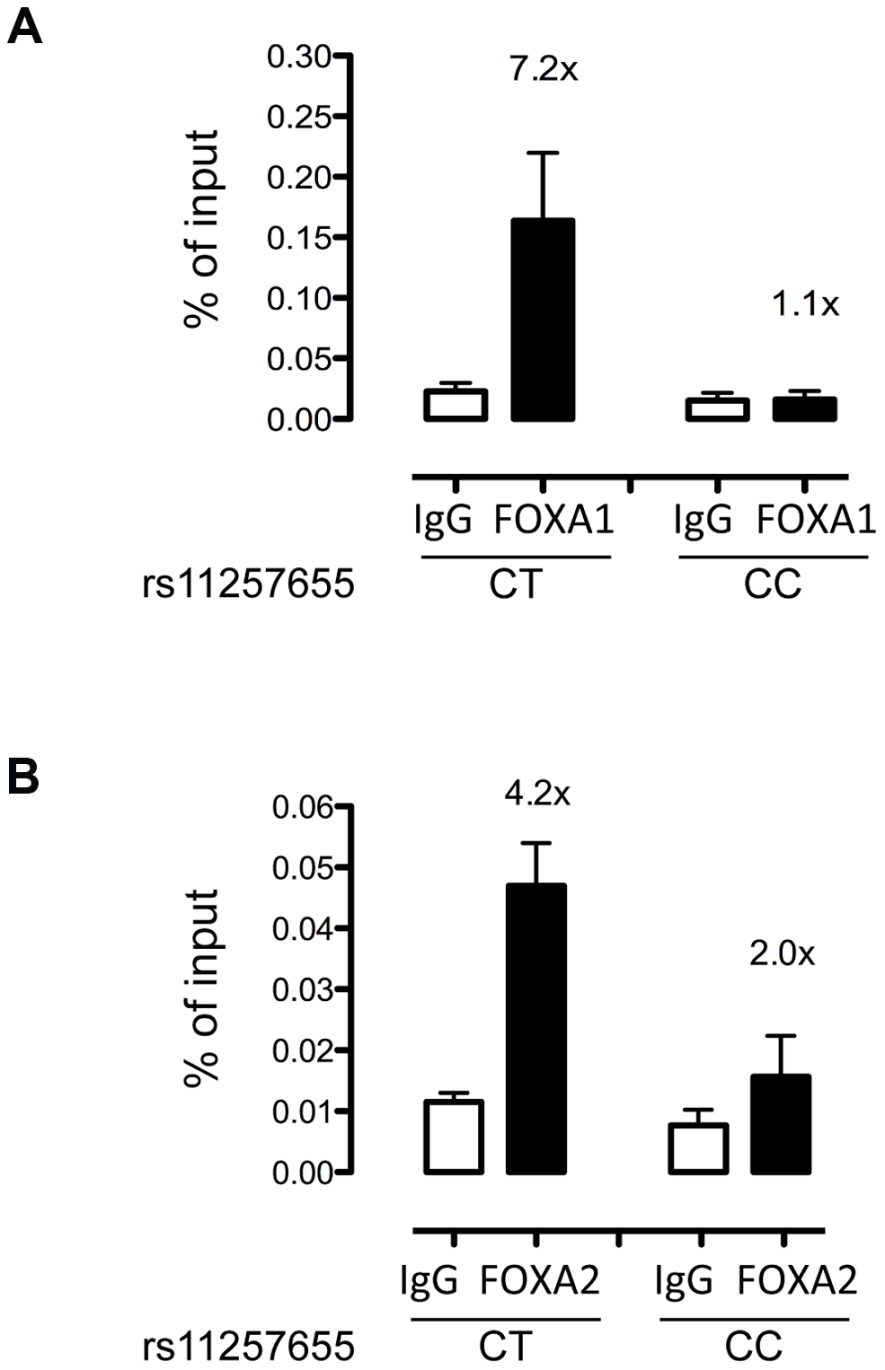
rs11257655-T allele shows increased binding to FOXA1 and FOXA2 in human islets. FOXA1 (A) and FOXA2 (B) ChIP in human islets shows enrichment at rs11257655 compared to IgG control. Islets containing one copy of the rs11257655-T allele show 7.2-fold greater FOXA1 enrichment and 4.2-fold greater FOXA2 enrichment. rs11257655 CT heterozygotes are more significantly enriched than rs11257655 CC homozygotes for FOXA1 (one-sided *t*-test, *P* = .06) and FOXA2 (one-sided *t*-test, *P* = .026). A negative control region 28 kb downstream of rs11257655 was not enriched in FOXA1- and FOXA2- bound chromatin (Figure S3A and S3B). Error bars represent standard error of two to three islets for each represented genotype.

To evaluate whether FOXA1 and FOXA2 bind differentially to rs11257655 in a native chromatin context, we performed allele-specific ChIP in human islets with different rs11257655 genotypes. FOXA1 was enriched 7.2-fold compared to IgG control in islets carrying a T allele while FOXA1 was not enriched in islets homozygous for C allele (Figure 5A). Although less robust, FOXA2 was enriched 4.2-fold in islets carrying a T allele compared to IgG control (Figure 5B). This direction of enrichment is consistent with the EMSA data (Figure 4). A region 28 kb downstream of rs11257655 with no evidence of open chromatin (chr10 control) was used as a negative control (Figure S3). These findings strengthen the conclusion that rs11257655 is part of a bona fide *cis*-regulatory complex binding FOXA1 and/or FOXA2 in human islets.

### *CDC123* and *CAMK1D* transcript levels

To determine whether *CDC123* or *CAMK1D* are expressed in type 2 diabetes-relevant tissues, we measured and confirmed expression of both transcripts in human islets and hepatocytes (Figure S4A, S4B). These data are supported by RNA-seq evidence that both genes are expressed in islets [24]. Based on our results showing islet beta cells as a target tissue of risk variant regulatory activity, we assessed whether glucose treatment regulated *CDC123* and *CAMK1D* transcript level. Glucose-mediated transcriptional changes in one of these genes might point to the more plausible candidate important in beta cell biology. In MIN6 cells treated with low (3 mM) and high (20 mM) concentrations of glucose for 16 hours, *CAMK1D* expression increased (P = .004; Figure S4C) while *CDC123* expression remained unchanged (P = .22; Figure S4D). In 832/13 cells, *CDC123* levels were significantly higher in cells stimulated with high glucose (P = 1.6×10^−5^; Figure S4E). We could not assess the effect of glucose on *CAMK1D* levels in 832/13 cells because this transcript level was below detection limits. While we confirm expression of *CAMK1D* and *CDC123* in islets and hepatocytes, future studies over-expressing the target gene(s) in these tissues would be necessary to establish the mechanisms by which increased expression leads to diabetes risk.

## Discussion

Integration of genome-wide regulatory annotation maps with disease-associated variants identified through GWAS has great potential for elucidation of gene-regulatory variants underlying association signals. In this study, we expand the lexicon of disease-associated functional regulatory variation by examining the type 2 diabetes-association signal at the *CDC123*/*CAMK1D* locus. We prioritized candidate *cis*-regulatory variants and tested whether prioritized variants exhibited allele-specific transcriptional enhancer activity. We provide transcriptional reporter and protein-DNA binding evidence that rs11257655 is part of a *cis*-regulatory complex differentially affecting transcriptional activity. Additionally, we validate FOXA1 and FOXA2 as components of this regulatory complex in human islets.

In recent years, progress has been made in following up mechanistic studies of GWAS type 2 diabetes-association signals [6], [7], [9], [25]–[30], but challenges remain in sifting through the many associated variants at a locus to identify those influencing disease. We hypothesized that a common variant with modest effect underlies the association at the *CDC123/CAMK1D* locus and evaluated the location of high LD variants (r^2^≥.7; n = 11) at the locus relative to known transcripts and to putative DNA regulatory elements. We identified two variants that overlapped putative islet and/or liver regulatory regions and none located in exons. We did not assess variants in lower LD (r^2^<.7), and additional functional SNPs may exist at this locus acting through alternate functional mechanisms untested in the current study.

Based on our observation of type 2 diabetes-associated SNPs in regions of islet and liver open chromatin, we measured transcriptional activity in two mammalian islet cell models, rat 832/13 and mouse MIN6 insulinoma cells and in one hepatocyte cell model, human HepG2 hepatocellular carcinoma cells. In agreement with our previous observations [7], we found good concordance in allelic transcriptional activity of human regulatory elements across the two rodent islet cell types. Of the two SNPs predicted to be located in predicted enhancer regions, rs11257655 but not rs36062557 demonstrated allele-specific effects in islets and liver, suggesting that rs11257655 is a lead functional candidate. The rs11257655-T allele associated with type 2 diabetes risk displayed increased enhancer activity relative to the C allele, suggesting that increased expression of one or more genes, possibly *CAMK1D* or *CDC123*, may be associated with type 2 diabetes. Our subsequent analysis of protein binding revealed complexes that favored the rs11257655-T allele in 832/13, MIN6 and HepG2 cells. Consistent with predictions that the rs11257655-C allele may disrupt binding to the FOXA1 and FOXA2 transcription factors, we demonstrated that only the T allele of rs11257655 leads to FOXA1- and FOXA2-mediated supershifts. The ChIP enrichment of FOXA1 and FOXA2 in human islets from carriers of the T allele is concordant with EMSAs using nuclear extract from mouse and rat cell lines, further demonstrating the utility of rodent islet cell models to characterize human regulatory elements. Our results suggest that a *cis*-regulatory element surrounding rs11257655 may act in both islet and liver cells. Although we provide evidence that rs11257655 alleles differentially bind *FOXA1* and *FOXA2 in vivo*, it is important to note that this enrichment was detected in isolated human islets. Future experiments will be needed to validate effects of rs11257655 within a whole organism environment. For example, recently zebrafish have been used to assay the regulatory potential of DNA sequences [31], [32].

FOXA1 and FOXA2 are members of the FOXA subclass of the forkhead box transcription factor family and are essential transcriptional activators in development of endodermally-derived tissues including liver and pancreas [33], [34]. In mature mouse β-cells, ablation of both transcription factors compared to ablation of FoxA2 alone leads to more pronounced impaired glucose homeostasis and insulin secretion, indicating that both factors are important in maintenance of the mature beta cell phenotype [35]. In addition, FoxA2 integrates the transcriptional response of mouse adult hepatocytes to a state of fasting [36]. FOXA1 and FOXA2 are thought to act as pioneer transcription factors, scanning chromatin for enhancers with forkhead motifs and opening compacted chromatin through DNA demethylation and subsequent induction of H3K4 methylation, epigenetic changes that likely render enhancers transcriptionally competent by allowing subsequent recruitment of transcriptional effectors [37]–[39]. Our data demonstrate increased transcriptional activity and increased binding of FOXA1 and FOXA2 to the rs11257655-T allele, suggesting that rs11257655 may be functioning as part of a transcriptional activator complex. Recent experiments in pancreatic islets support a role for FOXA transcription factors in activation of islet enhancers [40]. This same study also showed that FOXA2 binds in pancreatic islets in the T2D-associated region surrounding rs11257655. Further experiments, such as ChIP-seq of additional transcription factors, may identify other key factors present in the activator complex.

Both *CAMK1D* and *CDC123* are candidate transcripts affected by variation at this locus. C*is*-eQTLs in both blood and lung support an effect on *CAMK1D* but not *CDC123*. In blood, initial eQTL evidence for both genes were further analyzed by conditional analyses on the T2D lead SNP or rs11257655. The conditional analyses abolished the *cis*-eQTL signal for *CAMK1D* but not for *CDC123*, providing evidence that the T2D GWAS signal and the *CAMK1D cis*-eQTL signal are coincident [18]. In lung, the GTEx consortium identified an eQTL for *CAMK1D* with rs11257655 as a lead associated variant (*P* = 1.1×10^−7^); this and other T2D GWAS variants are the strongest cis-eQTLs for *CAMK1D*, while no significant eQTL is observed for *CDC123* [19]. For both eQTLs, the rs11257655 type 2 diabetes risk allele is associated with increased *CAMK1D* transcript level, consistent with the direction of transcriptional activity we observed for this allele in islet and liver cells. Many eQTLs are predicted to be shared among tissues [41], and a recent study of the beta cell transcriptome reports good concordance of eQTL direction (R^2^ = .74–.76) between beta cells and blood-derived lymphoblastoid cell lines, fat and skin [42], suggesting that the *CAMK1D* eQTL may also exist in islets. Some eQTLs differ across tissues, and evidence of a consistent eQTL in islets would be valuable. Knockout mice provide further evidence supporting *CAMK1D* as a target gene. In *FoxA1/FoxA2* beta cell-specific knockout mice, *Camk1d* expression was reported to be slightly reduced (1.8 fold, *P* = 0.13) [35], consistent with our conclusion that rs11257655 is part of a transcriptional activator complex that includes FOXA1 and FOXA2. Together, these data suggest that *CAMK1D* is a more plausible target for differential regulation by rs11257655 alleles.

The mechanism by which *CAMK1D* may act in type 2 diabetes biology is unclear. CAMK1D is a serine threonine kinase that operates in the calcium-triggered CaMKK-CaMK1 signaling cascade [17], [43]. In response to calcium influx, *CAMK1D* activates CREB- (cAMP response element-binding protein) dependent gene transcription by phosphorylation [17]. CREB is a key beta cell regulator important in glucose sensing, insulin exocytosis and gene transcription and β-cell survival [44], and FOXA2 has been shown to be necessary to mediate recruitment of CREB in fasting-induced activation of hepatic gluconeogenesis [36]. *CAMK1D* also has been reported to regulate glucose in primary human hepatocytes [45]. It is important to note that we cannot rule out cell cycle regulator *CDC123* as a target for regulation by rs11257655.

In conclusion, we extend follow up studies of GWAS-identified type 2 diabetes-associated variants to the *CDC123/CAMK1D* locus on chromosome 10. We identify rs11257655 as part of a *cis* regulatory complex in islet and liver cells that alters transcriptional activity through binding FOXA1 and FOXA2. These data demonstrate the utility of experimentally predicted chromatin state to identify regulatory variants for complex traits.

## Materials and Methods

### Selection of SNPs for functional study

Variants were prioritized for functional study based on linkage disequilibrium (LD) and evidence of being in an islet or liver regulatory element based on data from the ENCODE consortium [46]. Of 11 variants meeting the LD threshold (r^2^≥.7, EUR, with the GWAS index SNP rs12779790, 1000G Phase 1 release), two SNPs showed evidence of open chromatin [6], [9], [20], [47], histone modifications [21], [22], [48] or transcription factor binding and were tested for evidence of differential transcriptional activity.

### Cell culture

Two insulinoma cell lines, rat-derived 832/13 [49] (C.B. Newgard, Duke University) and mouse-derived MIN6 [50] were maintained at 37°C with 5% CO_2_. 832/13 cells were cultured in RPMI 1640 (Cellgro/Corning) supplemented with 10% FBS, 1 mM sodium pyruvate, 2 mM L-glutamine, 10 mM HEPES and 0.05 mM β-mercaptoethanol. MIN6 cells were cultured in DMEM (Sigma), supplemented with 10% FBS, 1 mM sodium pyruvate, 0.1 mM β-mercaptoethanol. HepG2 hepatocellular carcinoma cells were cultured in MEM-alpha (Gibco) supplemented with 10% FBS, 1 mM sodium pyruvate and 2 mM L-glutamine.

### Generation of luciferase reporter constructs, transient DNA transfection and luciferase reporter assays

Fragments surrounding each of rs11257655 (151 bp) and rs34428576 (179 bp) were PCR-amplified (Table S1) from DNA of individuals homozygous for risk and non-risk alleles. Restriction sites for KpnI and XhoI were added to primers during amplification, and the resulting PCR products were digested with KpnI and XhoI and cloned in both orientations into the multiple cloning site of the minimal promoter-containing firefly luciferase reporter vector pGL4.23 (Promega, Madison, WI). Fragments are designated as ‘forward’ or ‘reverse’ based on their orientation with respect to the genome. Two to five independent clones for each allele for each orientation were isolated, verified by sequencing, and transfected in duplicate into 832/13, MIN6 and HepG2 cell lines. Missing haplotypes of rs36062557-rs11257655 constructs were created using the QuikChange site directed mutagenesis kit (Stratagene).

Approximately 1×10^−5^ cells per well were seeded in 24-well plates. At 80% confluency, cells were co-transfected with luciferase constructs and *Renilla* control reporter vector (phRL-TK, Promega) at a ratio of 10∶1 using Lipofectamine 2000 (Invitrogen) for 832/13, and using FUGENE-6 for MIN6 and HepG2 cells (Roche Diagnostics, Indianapolis, IN). 48 h after transfection, cells were lysed with passive lysis buffer (Promega), and luciferase activity was measured using the Dual-luciferase assay system (Promega). To control for transfection efficiency, raw values for firefly luciferase activity were divided by raw *Renilla* luciferase activity values, and fold change was calculated as normalized luciferase values divided by pGL4.23 minimal promoter empty vector control values. Data are reported as the fold change in mean (± SD) relative luciferase activity per allele. A two-sided *t*-test was used to compare luciferase activity between alleles. All experiments were carried out on a second independent day and yielded comparable allele-specific results.

### Electrophoretic mobility shift assay (EMSA)

Nuclear cell extracts were prepared from 832/13, MIN6, and HepG2 cells using the NE-PER nuclear and cytoplasmic extraction kit (Thermo Scientific) according to the manufacturer's instructions. Protein concentration was measured with a BCA protein assay (Thermo Scientific), and lysates were stored at −80°C until use. 21 bp oligonucleotides were designed to the sequence surrounding rs11257655 risk or non-risk alleles: Sense 5′ biotin- GGGCAAGTGT[**C/T**]TACTGGGCAT 3′, antisense 5′ biotin- ATGCCCAGTA[**G/A**]ACACTTGCCC 3′ (SNP allele in bold). Double-stranded oligonucleotides for the risk and non risk alleles were generated by incubating 50 pmol complementary oligonucleotides at 95°C for 5 minutes followed by gradual cooling to room temperature. EMSA's were carried out using the LightShift Chemiluminescent EMSA Kit (Thermo Scientific). Binding reactions were set up as follows: 1× binding buffer, 50 ng/µL poly (dI•dC), 3 µg nuclear extract, 200 fmol of labeled probe in a final volume of 20 µL. For competition reactions, 67-fold excess of unlabeled double-stranded oligonucleotides for either the risk or non-risk allele were included. Reactions were incubated at room temperature for 25 minutes. For supershift assays, 4 µg of polyclonal antibodies against FOXA1 (ab23738; Abcam) or FOXA2 (SC6554X; Santa Cruz Biotechnology) was added to the binding reaction and incubation proceeded for a further 25 minutes. Binding reactions were subjected to non-denaturing PAGE on DNA retardation gels in 0.5× TBE (Lonza), transferred to Biodyne nylon membranes (Thermo Scientific) and cross-linked on a UV-light cross linker (Stratagene). Biotin labeled DNA-protein complexes were detected by chemiluminescence. EMSAs were carried out on a second independent day and yielded comparable.

### DNA affinity capture assay

DNA affinity capture was carried out as previously described [7]. Briefly, dialyzed nuclear extracts (300 µg) were pre-cleared with 100 µl of streptavidin-agarose dynabeads (Invitrogen) coupled to biotin-labeled scrambled control oligonucleotides. For DNA-protein binding reactions, 40 pmol of biotin labeled probe for either rs11257655 allele (same probe as for EMSA) or for a scrambled control were incubated with 300 µg nuclear extract, binding buffer (10 mM Tris, 50 mM KCL, 1 mM DTT), 0.055 µg/µL poly (dI•dC) and H_2_0 to total 450 µL at room temperature for 30 minutes with rotation. 100 µL (1 mg) of streptavidin-agarose dynabeads were added and the reaction incubated for a further 20 minutes. Beads were washed and DNA-bound proteins were eluted in 1× reducing sample buffer (Invitrogen). Proteins were separated on NuPAGE denaturing gels and protein bands stained with SYPRO-Ruby. Protein bands displaying differential binding between rs11257655 alleles were excised from the gel and subjected to matrix assisted laser desorption time-of-flight/time-of-flight tandem mass spectrometry (MS) and analysis at the University of North Carolina proteomics core facility. For peptide identification, all MS/MS spectra were searched against all entries, NCBI non-redundant (NR) database, using GPS Explorer Software Version 3.6 (ABI) and the Mascot (MatrixScience) search algorithm. Mass tolerances of 80 ppm for precursor ions and 0.6 Da for fragment ions were used. In addition, two missed cleavages were allowed and oxidation of methionine was a variable modification.

### Chromatin Immunoprecipitation (ChIP) assays

Human islets from non-diabetic organ donors were provided by the National Disease Research Interchange (NDRI). Use of human tissues was approved by the University of North Carolina Institutional Review Board. Islet viability and purity were assessed by the NDRI. Islets were warmed to 37°C and washed with calcium- and magnesium-free Dulbecco's phosphate-buffered saline (Life Technologies) prior to crosslinking. For chromatin immunoprecipitation (ChIP) studies, approximately 2000 islet equivalents (IEQs) were crosslinked for 10 min in 1% formaldehyde (Sigma-Aldrich) at room temperature. Islets were lysed and chromatin was sheared on ice using a standard bioruptor (Diagenode; 20–22 cycles of 30 s sonication with 1 min rest between cycles) to a size of 200–1000 bp. IP dilution buffer (0.01% SDS, 1.1% Triton X-100, 1.2 mM EDTA, 16.7 mM Tris at pH 8.1, 167 mM NaCl, protease inhibitors) was added, 5% of the volume was removed and used as input, and the remainder was incubated overnight at 4°C on a nutating platform with FOXA1 or FOXA2 antibody or a species-matched IgG as control. Antibodies used for ChIP were the same as for EMSA; FOXA1 (Abcam) and FOXA2 (Santa Cruz). Protein A agarose beads (Santa Cruz) were added and incubated for 3 h at 4°C. Beads were then washed for 5 minutes at 4°C with gentle mixing, using the following solutions: Low Salt Buffer (0.1% SDS, 1% Triton X-100, 2 mM EDTA, 20 mM Tris, 150 mM NaCl); High Salt Buffer (0.1% SDS, 1% Triton X-100, 2 mM EDTA, 20 mM Tris, 500 mM NaCl); LiCl buffer (1 mM EDTA, 10 mM Tris, 250 mM LiCl, 1% NP-40, 1% Na-Deoxycholate), twice; and TE buffer (Sigma-Aldrich), twice. Chromatin was eluted from beads with two 15-minute washes at 65°C using freshly prepared Elution Buffer (1% SDS/0.1 M NaHCO_3_). To reverse crosslinks, 5 M NaCl was added to each sample to a final concentration of 0.2 M, and incubated overnight at 65°C; to remove protein, samples were incubated with 10 uL 0.5 M EDTA, 20 uL 1 M Tris (pH 6.5) and 3 uL of Proteinase K (10 mg/mL) at 45°C for 3 hours. DNA was extracted with 25∶24∶1 phenol:choloform:isoamyl alcohol, precipitated with 100% ethanol with 1 µl glycogen as a carrier, and resuspended in TE (Sigma). qPCR was performed in triplicate using SYBR Green Master Mix. Primers were designed to amplify a 99-bp region surrounding rs112576555; 5′-CTACTGCTTCTCCGGACTCG ′3′ and 5′- TGGCCTCAAGAGG GAGATAA -3′. Primers for a 133-bp control region not overlapping open chromatin and located 27 kb away were 5′-GCACCCATGGTACTGAAACC -3′ and 5′- CTTTTCCCG AGGAAGGAACT -3′. Dissociation curves demonstrated a single PCR product in each case without primer dimers. Fold enrichment was calculated as FOXA1/FOXA2 enrichment divided by IgG control. A one-sided t-test was performed to compare enrichment based on the direction of binding observed using EMSA.

### Effect of glucose on *Cdc123* and *Camk1d* transcript level

To measure effects of glucose on expression of *Cdc123* and *Camk1d*, 832/13 cells and MIN6 cells were washed with PBS and preincubated for 2.0 h in secretion buffer (114 mm NaCl, 4.7 mm KCl, 1.2 mm KH_2_PO_4_, 1.16 mm MgSO_4_, 20 mm HEPES, 2.5 mm CaCl_2_, 0.2% BSA, pH 7.2. For GSIS, cells were incubated in secretion buffer for an additional 2 hours or 16 hours in the presence of 3 mM or 20 mM glucose and then harvested for RNA.

### RNA isolation and quantitative real-time reverse-transcription PCR

Total cytosolic RNA was isolated using the RNeasy Mini Kit (Qiagen). RNA concentrations were determined using a Nanodrop 1000 (Thermo Scientific, Wilmington, DE, USA). For real-time reverse transcription (RT)–PCR, first-strand cDNA was synthesized using 8 ul of total RNA in a 20 µl reverse transcriptase reaction mixture (Superscript III First strand synthesis kit; Life Technologies). cDNA was diluted to contain equivalent to 20–55 ng/µl input RNA. To measure total human mRNA levels of *CDC123*, *CAMK1D* and *B2M*, gene-specific primers and fast SYBR Green Master Mix (Life Technologies) were used (Table S2). TaqMan designed gene expression assays (Life Technologies) were used to measure *Cdc123*, *Camk1D* and *Rsp9* (housekeeping gene) mRNA levels of mouse and rat cells. All PCR reactions were performed in triplicate in a 10-µl volume using a STEPOne Plus real-time PCR system (Life Technologies). Serial 3-fold dilutions of cDNA from pooled human tissues, 832/13 or MIN6 cells as appropriate were used as a reference for a standard curve. Statistical significance was determined by two-tailed *t*-tests.

## Supporting Information

Figure S1 Regulatory potential at rs11257655 and rs36062557. UCSC genome browser (hg18) diagram showing that rs11257655 and rs36062557 overlap regions of open chromatin, detected by DNase hypersensitivity and FAIRE, and histone modifications, including H3K4me1 and H3K9ac in islet, liver, and HepG2 cells. H3K27ac and H3K4me3 histone modifications are also shown. rs11257655 and rs36062557 are also located near to HepG2 ChIP-seq peaks for FOXA1 and FOXA2. DNA sequences amplified to evaluate transcriptional activity in dual-luciferase reporter assays and to evaluate enrichment of binding to FOXA1 and FOXA2 are indicated.
(TIF)

Figure S2 Transcriptional activity at rs34428576. Enhancer activity was measured in 832/13 cells (A) and HepG2 cells (B) for alleles of rs34428576. No difference was observed between alleles in 832/13 cells. In HepG2 cells, moderate allele-specific activity was observed only in the reverse orientation. Error bars represent standard deviation of 4–5 independent clones for each allele. Results are expressed as fold change compared to empty vector control. *P* values were calculated by a two-sided t-test.
(TIF)

Figure S3 Chromosome 10 region not overlapping open chromatin does not show binding to FOXA1 and FOXA2 in human islets. A negative control region 28 kb downstream of rs11257655 was not substantially enriched in FOXA1- (A) and FOXA2- (B) bound chromatin. Error bars represent standard error of two to three islets for each represented genotype.
(TIF)

Figure S4 *CDC123* and *CAMK1D* expression and response to glucose. (A, B) Evidence that *CAMK1D* and *CDC123* are expressed in various human tissues. cDNA from human islets, hepatocytes, blood and adipocytes was analyzed by real-time PCR using gene-specific primers for *CAMK1D* (A) and *CDC123 and B2M* (B). mRNA level was normalized to *B2M*. (C, D, E) Effect of glucose stimulus on *CAMK1D* and *CDC123* expression level. 832/13 and MIN6 insulinoma cells were treated with low (3 mM) and high (15 mM) glucose for 16–18 hours. cDNA was analyzed by real-time PCR using *TaqMan gene expression assays for CAMK1D* (C) and *CDC123 (D, E). mRNA level was normalized to RSP9. High glucose treatment resulted in a significant increase in CAMK1D mRNA level (C) but not CDC123 in MIN6 cells (D). High glucose treatment resulted in increased CDC123 mRNA level in 832/13 cells*. Error bars represent the standard deviation of 4–5 samples for each treatment. *P* values were calculated by a two-sided *t*-test.
(TIF)

Table S1 DNA sequences amplified for luciferase activity assays.
(DOCX)

Table S2 PCR primers for quantitative real-time PCR in human tissues.
(DOCX)

## References

[1] Poulsen P, Kyvik KO, Vaag A, Beck-Nielsen H (1999) Heritability of type II (non-insulin-dependent) diabetes mellitus and abnormal glucose tolerance–a population-based twin study. Diabetologia 42: 139–145. 10064092 10.1007/s001250051131

[2] Morris AP, Voight BF, Teslovich TM, Ferreira T, Segre AV, et al (2012) Large-scale association analysis provides insights into the genetic architecture and pathophysiology of type 2 diabetes. Nat Genet 44: 981–990. 22885922 10.1038/ng.2383 PMC3442244

[3] Zeggini E, Scott LJ, Saxena R, Voight BF, Marchini JL, et al (2008) Meta-analysis of genome-wide association data and large-scale replication identifies additional susceptibility loci for type 2 diabetes. Nat Genet 40: 638–645. 18372903 10.1038/ng.120 PMC2672416

[4] Shu XO, Long J, Cai Q, Qi L, Xiang YB, et al (2010) Identification of new genetic risk variants for type 2 diabetes. PLoS Genet 6: e1001127. 20862305 10.1371/journal.pgen.1001127 PMC2940731

[5] Kooner JS, Saleheen D, Sim X, Sehmi J, Zhang W, et al (2011) Genome-wide association study in individuals of South Asian ancestry identifies six new type 2 diabetes susceptibility loci. Nat Genet 43: 984–989. 21874001 10.1038/ng.921 PMC3773920

[6] Stitzel ML, Sethupathy P, Pearson DS, Chines PS, Song L, et al (2010) Global epigenomic analysis of primary human pancreatic islets provides insights into type 2 diabetes susceptibility loci. Cell Metab 12: 443–455. 21035756 10.1016/j.cmet.2010.09.012 PMC3026436

[7] Fogarty MP, Panhuis TM, Vadlamudi S, Buchkovich ML, Mohlke KL (2013) Allele-specific transcriptional activity at type 2 diabetes-associated single nucleotide polymorphisms in regions of pancreatic islet open chromatin at the JAZF1 locus. Diabetes 62: 1756–1762. 23328127 10.2337/db12-0972 PMC3636602

[8] Paul DS, Albers CA, Rendon A, Voss K, Stephens J, et al (2013) Maps of open chromatin highlight cell type-restricted patterns of regulatory sequence variation at hematological trait loci. Genome Res 23: 1130–1141. 23570689 10.1101/gr.155127.113 PMC3698506

[9] Gaulton KJ, Nammo T, Pasquali L, Simon JM, Giresi PG, et al (2010) A map of open chromatin in human pancreatic islets. Nat Genet 42: 255–259. 20118932 10.1038/ng.530 PMC2828505

[10] Paul DS, Nisbet JP, Yang TP, Meacham S, Rendon A, et al (2011) Maps of open chromatin guide the functional follow-up of genome-wide association signals: application to hematological traits. PLoS Genet 7: e1002139. 21738486 10.1371/journal.pgen.1002139 PMC3128100

[11] Dimas AS, Lagou V, Barker A, Knowles JW, Magi R, et al (2013) Impact of type 2 diabetes susceptibility variants on quantitative glycemic traits reveals mechanistic heterogeneity. Diabetes 10.2337/db13-0949 PMC4030103 24296717

[12] Soler Artigas M, Loth DW, Wain LV, Gharib SA, Obeidat M, et al (2011) Genome-wide association and large-scale follow up identifies 16 new loci influencing lung function. Nat Genet 43: 1082–1090. 21946350 10.1038/ng.941 PMC3267376

[13] Bieganowski P, Shilinski K, Tsichlis PN, Brenner C (2004) Cdc123 and checkpoint forkhead associated with RING proteins control the cell cycle by controlling eIF2gamma abundance. J Biol Chem 279: 44656–44666. 15319434 10.1074/jbc.M406151200

[14] Perzlmaier AF, Richter F, Seufert W (2013) Translation initiation requires cell division cycle 123 (Cdc123) to facilitate biogenesis of the eukaryotic initiation factor 2 (eIF2). J Biol Chem 288: 21537–21546. 23775072 10.1074/jbc.M113.472290 PMC3724614

[15] Visscher PM, Brown MA, McCarthy MI, Yang J (2012) Five years of GWAS discovery. Am J Hum Genet 90: 7–24. 22243964 10.1016/j.ajhg.2011.11.029 PMC3257326

[16] Sakagami H, Kamata A, Nishimura H, Kasahara J, Owada Y, et al (2005) Prominent expression and activity-dependent nuclear translocation of Ca2+/calmodulin-dependent protein kinase Idelta in hippocampal neurons. Eur J Neurosci 22: 2697–2707. 16324104 10.1111/j.1460-9568.2005.04463.x

[17] Verploegen S, Lammers JW, Koenderman L, Coffer PJ (2000) Identification and characterization of CKLiK, a novel granulocyte Ca(++)/calmodulin-dependent kinase. Blood 96: 3215–3223. 11050006

[18] Voight BF, Scott LJ, Steinthorsdottir V, Morris AP, Dina C, et al (2010) Twelve type 2 diabetes susceptibility loci identified through large-scale association analysis. Nat Genet 42: 579–589. 20581827 10.1038/ng.609 PMC3080658

[19] Consortium GT (2013) The Genotype-Tissue Expression (GTEx) project. Nat Genet 45: 580–585. 23715323 10.1038/ng.2653 PMC4010069

[20] Song L, Zhang Z, Grasfeder LL, Boyle AP, Giresi PG, et al (2011) Open chromatin defined by DNaseI and FAIRE identifies regulatory elements that shape cell-type identity. Genome Res 21: 1757–1767. 21750106 10.1101/gr.121541.111 PMC3202292

[21] Creyghton MP, Cheng AW, Welstead GG, Kooistra T, Carey BW, et al (2010) Histone H3K27ac separates active from poised enhancers and predicts developmental state. Proc Natl Acad Sci U S A 107: 21931–21936. 21106759 10.1073/pnas.1016071107 PMC3003124

[22] Heintzman ND, Hon GC, Hawkins RD, Kheradpour P, Stark A, et al (2009) Histone modifications at human enhancers reflect global cell-type-specific gene expression. Nature 459: 108–112. 19295514 10.1038/nature07829 PMC2910248

[23] Bryne JC, Valen E, Tang MH, Marstrand T, Winther O, et al (2008) JASPAR, the open access database of transcription factor-binding profiles: new content and tools in the 2008 update. Nucleic Acids Res 36: D102–106. 18006571 10.1093/nar/gkm955 PMC2238834

[24] Parker SC, Stitzel ML, Taylor DL, Orozco JM, Erdos MR, et al (2013) Chromatin stretch enhancer states drive cell-specific gene regulation and harbor human disease risk variants. Proc Natl Acad Sci U S A 110: 17921–17926. 24127591 10.1073/pnas.1317023110 PMC3816444

[25] Rees MG, Wincovitch S, Schultz J, Waterstradt R, Beer NL, et al (2012) Cellular characterisation of the GCKR P446L variant associated with type 2 diabetes risk. Diabetologia 55: 114–122. 22038520 10.1007/s00125-011-2348-5 PMC3276843

[26] Beer NL, Tribble ND, McCulloch LJ, Roos C, Johnson PR, et al (2009) The P446L variant in GCKR associated with fasting plasma glucose and triglyceride levels exerts its effect through increased glucokinase activity in liver. Hum Mol Genet 18: 4081–4088. 19643913 10.1093/hmg/ddp357 PMC2758140

[27] Travers ME, Mackay DJ, Dekker Nitert M, Morris AP, Lindgren CM, et al (2013) Insights into the molecular mechanism for type 2 diabetes susceptibility at the KCNQ1 locus from temporal changes in imprinting status in human islets. Diabetes 62: 987–992. 23139357 10.2337/db12-0819 PMC3581222

[28] Nicolson TJ, Bellomo EA, Wijesekara N, Loder MK, Baldwin JM, et al (2009) Insulin storage and glucose homeostasis in mice null for the granule zinc transporter ZnT8 and studies of the type 2 diabetes-associated variants. Diabetes 58: 2070–2083. 19542200 10.2337/db09-0551 PMC2731533

[29] Lecompte S, Pasquetti G, Hermant X, Grenier-Boley B, Gonzalez-Gross M, et al (2013) Genetic and molecular insights into the role of PROX1 in glucose metabolism. Diabetes 62: 1738–1745. 23274905 10.2337/db12-0864 PMC3636631

[30] Ng HJ, Gloyn AL (2013) Bridging the gap between genetic associations and molecular mechanisms for type 2 diabetes. Curr Diab Rep 13: 778–785. 24127137 10.1007/s11892-013-0429-1

[31] Camp JG, Jazwa AL, Trent CM, Rawls JF (2012) Intronic cis-regulatory modules mediate tissue-specific and microbial control of angptl4/fiaf transcription. PLoS Genet 8: e1002585. 22479192 10.1371/journal.pgen.1002585 PMC3315460

[32] Ishibashi M, Mechaly AS, Becker TS, Rinkwitz S (2013) Using zebrafish transgenesis to test human genomic sequences for specific enhancer activity. Methods 62: 216–225. 23542551 10.1016/j.ymeth.2013.03.018

[33] Lee CS, Friedman JR, Fulmer JT, Kaestner KH (2005) The initiation of liver development is dependent on Foxa transcription factors. Nature 435: 944–947. 15959514 10.1038/nature03649

[34] Gao N, LeLay J, Vatamaniuk MZ, Rieck S, Friedman JR, et al (2008) Dynamic regulation of Pdx1 enhancers by Foxa1 and Foxa2 is essential for pancreas development. Genes Dev 22: 3435–3448. 19141476 10.1101/gad.1752608 PMC2607077

[35] Gao N, Le Lay J, Qin W, Doliba N, Schug J, et al (2010) Foxa1 and Foxa2 maintain the metabolic and secretory features of the mature beta-cell. Mol Endocrinol 24: 1594–1604. 20534694 10.1210/me.2009-0513 PMC2940470

[36] Zhang L, Rubins NE, Ahima RS, Greenbaum LE, Kaestner KH (2005) Foxa2 integrates the transcriptional response of the hepatocyte to fasting. Cell Metab 2: 141–148. 16098831 10.1016/j.cmet.2005.07.002

[37] Serandour AA, Avner S, Percevault F, Demay F, Bizot M, et al (2011) Epigenetic switch involved in activation of pioneer factor FOXA1-dependent enhancers. Genome Res 21: 555–565. 21233399 10.1101/gr.111534.110 PMC3065703

[38] Cirillo LA, Lin FR, Cuesta I, Friedman D, Jarnik M, et al (2002) Opening of compacted chromatin by early developmental transcription factors HNF3 (FoxA) and GATA-4. Mol Cell 9: 279–289. 11864602 10.1016/s1097-2765(02)00459-8

[39] Sekiya T, Muthurajan UM, Luger K, Tulin AV, Zaret KS (2009) Nucleosome-binding affinity as a primary determinant of the nuclear mobility of the pioneer transcription factor FoxA. Genes Dev 23: 804–809. 19339686 10.1101/gad.1775509 PMC2666343

[40] Pasquali L, Gaulton KJ, Rodriguez-Segui SA, Mularoni L, Miguel-Escalada I, et al (2014) Pancreatic islet enhancer clusters enriched in type 2 diabetes risk-associated variants. Nat Genet 46: 136–143. 24413736 10.1038/ng.2870 PMC3935450

[41] Flutre T, Wen X, Pritchard J, Stephens M (2013) A statistical framework for joint eQTL analysis in multiple tissues. PLoS Genet 9: e1003486. 23671422 10.1371/journal.pgen.1003486 PMC3649995

[42] Nica AC, Ongen H, Irminger JC, Bosco D, Berney T, et al (2013) Cell-type, allelic, and genetic signatures in the human pancreatic beta cell transcriptome. Genome Res 23: 1554–1562. 23716500 10.1101/gr.150706.112 PMC3759730

[43] Verploegen S, Ulfman L, van Deutekom HW, van Aalst C, Honing H, et al (2005) Characterization of the role of CaMKI-like kinase (CKLiK) in human granulocyte function. Blood 106: 1076–1083. 15840691 10.1182/blood-2004-09-3755

[44] Dalle S, Quoyer J, Varin E, Costes S (2011) Roles and regulation of the transcription factor CREB in pancreatic β -cells. Curr Mol Pharmacol 4: 187–195. 21488836 10.2174/1874467211104030187

[45] Haney S, Zhao J, Tiwari S, Eng K, Guey LT, et al (2013) RNAi screening in primary human hepatocytes of genes implicated in genome-wide association studies for roles in type 2 diabetes identifies roles for CAMK1D and CDKAL1, among others, in hepatic glucose regulation. PLoS One 8: e64946. 23840313 10.1371/journal.pone.0064946 PMC3688709

[46] Consortium EP, Bernstein BE, Birney E, Dunham I, Green ED, et al (2012) An integrated encyclopedia of DNA elements in the human genome. Nature 489: 57–74. 22955616 10.1038/nature11247 PMC3439153

[47] ENCODE_Project_Consortium (2011) A user's guide to the encyclopedia of DNA elements (ENCODE). PLoS Biol 9: e1001046. 21526222 10.1371/journal.pbio.1001046 PMC3079585

[48] Zhou X, Maricque B, Xie M, Li D, Sundaram V, et al (2011) The Human Epigenome Browser at Washington University. Nat Methods 8: 989–990. 22127213 10.1038/nmeth.1772 PMC3552640

[49] Hohmeier HE, Mulder H, Chen G, Henkel-Rieger R, Prentki M, et al (2000) Isolation of INS-1-derived cell lines with robust ATP-sensitive K+ channel-dependent and -independent glucose-stimulated insulin secretion. Diabetes 49: 424–430. 10868964 10.2337/diabetes.49.3.424

[50] Miyazaki J, Araki K, Yamato E, Ikegami H, Asano T, et al (1990) Establishment of a pancreatic beta cell line that retains glucose-inducible insulin secretion: special reference to expression of glucose transporter isoforms. Endocrinology 127: 126–132. 2163307 10.1210/endo-127-1-126

[51] Fujita PA, Rhead B, Zweig AS, Hinrichs AS, Karolchik D, et al (2011) The UCSC Genome Browser database: update 2011. Nucleic Acids Res 39: D876–882. 20959295 10.1093/nar/gkq963 PMC3242726

