# Supplementary material for: Identification of a Regulatory Variant That Binds FOXA1 and FOXA2 at the CDC123/CAMK1D Type 2 Diabetes GWAS Locus
Source: PLoS Genet. 2014 Sep 11;10(9):e1004633. doi: 10.1371/journal.pgen.1004633 (PMC4161327; doi:10.1371/journal.pgen.1004633)
Supplement: Table S1 — DNA sequences amplified for luciferase activity assays. (DOCX) [file pgen.1004633.s005.docx]

| SNP | Chromosome position (hg19) | Sequence 5’-3’ |
| --- | --- | --- |
| rs11257655 | chr10:12,307,791-12,307,941 | GGCCCAGAAATGACACAGAA |
|  |  | AACTGGGTAAGGCTCACTTCC |
| rs34428576 | chr10:12,280,974 -12,281,152 | GCGAGACTCTGCCTCAAAAG  GACAGAGTGAGACCCCATCC |

Restriction sites were added to primers for subcloning.
