# Supplementary material for: Identification of a Regulatory Variant That Binds FOXA1 and FOXA2 at the CDC123/CAMK1D Type 2 Diabetes GWAS Locus
Source: PLoS Genet. 2014 Sep 11;10(9):e1004633. doi: 10.1371/journal.pgen.1004633 (PMC4161327; doi:10.1371/journal.pgen.1004633)
Supplement: Table S2 — PCR primers for quantitative real-time PCR in human tissues. (DOCX) [file pgen.1004633.s006.docx]

| Gene | Sequence 5’-3’ |
| --- | --- |
| *CAMK1D* | Fwd – ATCTCCACAGAATGGGCATC |
|  | Rvs – CAGTTCAACGGCTTTGCTGTA |
| *CDC123* | Fwd – GCAGCTGGAGGATGAAGAAG |
|  | Rvs –TCATCCCTTCCTGAAACCAC |
| *B2M* | Fwd –TGTCTGGGTTTCATCCATCCGACA |
|  | Rvs –TCACACGGCAGGCATACTCATCTT |

**Figure S1: Regulatory potential at rs11257655 and rs36062557**

UCSC genome browser (hg18) diagram showing that rs11257655 and rs36062557 overlap regions of open chromatin, detected by DNase hypersensitivity and FAIRE, and histone modifications, including H3K4me1 and H3K9ac in islet, liver, and HepG2 cells. H3K27ac and H3K4me3 histone modifications are also shown. rs11257655 and rs36062557 are also located near to HepG2 ChIP-seq peaks for FOXA1 and FOXA2. DNA sequences amplified to evaluate transcriptional activity in dual-luciferase reporter assays and to evaluate enrichment of binding to FOXA1 and FOXA2 are indicated.

**Figure S2: Transcriptional activity at rs34428576**

Enhancer activity was measured in 832/13 cells (A) and HepG2 cells (B) for alleles of rs34428576. No difference was observed between alleles in 832/13 cells. In HepG2 cells, moderate allele-specific activity was observed only in the reverse orientation. Error bars represent standard deviation of 4-5 independent clones for each allele. Results are expressed as fold change compared to empty vector control. *P* values were calculated by a two-sided t-test.

**Figure S3: Chromosome 10 region not overlapping open chromatin does not show binding to FOXA1 and FOXA2 in human islets**

A negative control region 28 kb downstream of rs11257655 was not substantially enriched in FOXA1- (A) and FOXA2- (B) bound chromatin. Error bars represent standard error of two to three islets for each represented genotype**.**

**Figure S4: *CDC123* and *CAMK1D* expression and response to glucose.**

(A, B) Evidence that *CAMK1D* and *CDC123* are expressed in various human tissues. cDNA from human islets, hepatocytes, blood and adipocytes was analyzed by real-time PCR using gene-specific primers for CAMK1D (**A**) and CDC123 and B2M (**B**). mRNA level was normalized to B2M. (C, D, E) Effect of glucose stimulus on *CAMK1D* and *CDC123* expression level. 832/13 and MIN6 insulinoma cells were treated with low (3 mM) and high (15 mM) glucose for 16 – 18 hours. cDNA was analyzed by real-time PCR using TaqMan gene expression assays for CAMK1D (**C**) and CDC123 (D, E). mRNA level was normalized to RSP9. High glucose treatment resulted in a significant increase in CAMK1D mRNA level (C) but not CDC123 in MIN6 cells (D). High glucose treatment resulted in increased CDC123 mRNA level in 832/13 cells. Error bars represent the standard deviation of 4-5 samples for each treatment. *P* values were calculated by a two-sided t-test.
